# Exploring the role of lifetime brain maturation trajectories and their determinants in the onset of psychiatric disorders

**DOI:** 10.1192/j.eurpsy.2023.47

**Published:** 2023-07-19

**Authors:** E. Maggioni

**Affiliations:** Dept. of Electronics Information and Bioengineering, Politecnico di Milano, Milan, Italy

## Abstract

**Abstract:**

Recently, the diffusion of sophisticated neuroimaging techniques has tremendously advanced our understanding of brain structure and function. Nevertheless, **the current knowledge of the neurobiology of complex mental illnesses** - like major psychoses and depression - **is limited, hindering the development and validation of biomarkers for diagnosis, prognosis, and prediction of treatment response**.

Increasing evidence is suggesting a crucial role of environmental, personal, and behavioral processes, interacting among themselves and with genetics, in shaping mental functioning and psychopathological risk. In this context, the study of brain maturation trajectories and of their association with genetic and environmental factors can provide key insights on the risk for the emergence of mental illnesses over lifetime.

The present lecture will provide an **overview of our recent research on the brain underpinnings of psychotic and affective disorders onsetting during either adolescence/young adulthood or late adulthood**. Evidence obtained from young samples of twins will be presented to provide useful information on the **genetic and environmental determinants of physiological and pathological neurodevelopmental trajectories.** The complex **relationships among life events, brain morphology and connectivity, and psychopathology** will be discussed by showing our recent findings on multicentric transdiagnostic samples of young adults and elders. Special focus will be given to the brain mechanisms affected by social stressors, including discrimination and bullyism, as well as chronic stress, and their possible role in facilitating the onset or in enhancing the severity of psychotic and affective disorders.

**Disclosure of Interest:**

None Declared

